# Comparing the impact of in-person vs. virtual 10-week family-based childhood obesity management program on anthropometric, cardiometabolic, and mental health outcomes

**DOI:** 10.3389/fped.2025.1669107

**Published:** 2025-11-17

**Authors:** Alexandra J. Heidl, Daisy Sun, Claudia Faustini, Madelaine Gierc, Andy Bains, Tamara R. Cohen

**Affiliations:** 1 Faculty of Land and Food Systems, The University of British Columbia, Vancouver, BC, Canada; 2 Michael G. DeGroote School of Medicine, McMaster University, Hamilton, ON, Canada; 3 Department of Mathematics and Statistics, Concordia University, Montreal, QC, Canada; 4 Generation Health Clinic, British Columbia Children's Hospital, Vancouver BC, Canada; 5 BC Children’s Hospital Research Institute, Healthy Starts, Vancouver, BC, Canada

**Keywords:** pediatric obesity, health behaviours, virtual program, cardiometabolic markers, BYI-2, intensive health behavior and lifestyle treatment

## Abstract

**Objectives:**

To compare differences in patient outcomes in individuals living with overweight and obesity who attended a 10-week multidisciplinary program delivered virtually vs. participants who completed the program in-person.

**Methods:**

Data from 27 youth (8–18 years old) who attended virtual programming were matched by sex and body mass index (BMI) z-scores to youth who completed in-person programming. Changes in anthropometric, biomarkers (glucose, lipids and liver enzymes) and mental health (Beck Youth Inventories, Second Edition; “BYI-2”) were compared across groups.

**Results:**

BMI z-scores did not differ between groups (*p* = 0.88). Cardiometabolic measurements showed no significant differences at 10-weeks for any parameter, and there were no significant differences in BYI-2T-scores across groups (*p* > 0.05).

**Conclusion:**

The mode of delivery of a pediatric multidisciplinary family-based program was not associated with significant differences in participant outcomes, suggesting that both modes of delivery are effective.

## 1 Introduction

Family-centered interventions provided by a multidisciplinary team remain the first line approach for managing obesity in children and adolescents (1). Generation Health Clinic (formerly Shapedown BC) is a hospital-based pediatric obesity management program that is funded by the Government of British Columbia (Canada) and delivered in partnership with local health authorities. The program is a 10-week multidisciplinary, family-centered group intervention that includes content on nutrition, movement behaviours, family dynamics, mental health, and behaviour change (2). Similar to other programs (3), a previous evaluation of Shapedown BC showed that participants experienced modest reductions in body mass index (BMI) z-scores and improvements in dietary habits and physical activity levels, alongside positive changes in self-concept and overall well-being (2).

Prior to the COVID-19 pandemic, Shapedown BC was exclusively offered as an in-person intervention. However, the pandemic caused interruptions to non-urgent pediatric health care, impeding access to programs like Shapedown BC. To align with provincial COVID-19 health and safety guidelines, the program shifted to a virtual format. Nuss et al. (4) found that transitioning from a blended to a virtual *community*-based early intervention program in BC during COVID-19 was as effective in improving child lifestyle behaviours and parental support-related behaviours. However, it is not known if Shapedown BC, a *hospital*-delivered program which serves children/adolescents with significant health complications, would yield the same results. Therefore, this study aimed to assess the impact of program delivery modality (in-person vs. virtual) on clinical outcomes, including differences in anthropometric, cardiometabolic, and mental health parameters between participants who attended Shapedown BC virtually during the COVID-19 pandemic to participants who completed the in-person program before the pandemic.

## 2 Methods

### 2.1 Inclusion and exclusion criteria

Children and adolescents (6–17 years old) were eligible for referral to Shapedown BC if they (i) have a BMI for age >85th percentile and present with one or more obesity-related complications (e.g., types 2 diabetes, sleep disordered breathing) or (ii) have a BMI for age >97th percentile. Between September 2021 and March 2022, a total of 27 families enrolled in the BC Children's Hospital (“BCCH”) Shapedown BC virtual program provided informed written assent (children/adolescents) and consent (parents/guardians) for researchers to conduct a chart review. Participants without informed written consent and assent were excluded from the study. Baseline data were matched by age, sex, and BMI z-scores for participants who completed the in-person program from 2016 to 2019 at BCCH. Three in-person participants were matched to more than one virtual participant.

### 2.2 Ethics

This study was conducted according to the guidelines laid down in the Declaration of Helsinki and all procedures involving research study participants were approved by the University of British Columbia Children and Women's Research Ethics Board (H21-02932).

### 2.3 Measures

Clinicians measured patients’ weight and height to calculate BMI and BMI z-scores (5). Cardiometabolic parameters were assessed at BCCH using standard procedures and included: triglycerides, fasting glucose, high-density lipoprotein cholesterol (“HDL-C”), low-density lipoprotein cholesterol (“LDL-C”), total cholesterol, total cholesterol ratio, and liver enzymes [alanine aminotransferase (“ALT”) and aspartate aminotransferase (“AST”)].

Demographic questionnaires, which included age and sex, were completed by either parent (for children ≤12 years) or adolescents (13–18 years). Mental health (anxiety and depressive symptoms, self-concept) was assessed using the Beck Youth Inventories, Second Edition (“BYI-2”) (6). Age- and sex-adjusted T-scores >55 are indicative of elevated anxiety and depression, while T-scores < 44 are indicative of lower than average self-concept.

### 2.4 Statistical analysis

All analyses were conducted using SAS version 9.4 (SAS Institute, Cary, NC), with significance set at *p* < 0.05. Data are presented as mean and standard deviation. Between-group differences were assessed using two-sample *t*-tests for normally distributed data and Mann–Whitney *U*-tests for non-parametric data. Within-group differences were evaluated using paired-sample *t*-tests (parametric) and Wilcoxon Signed-Rank tests (non-parametric). Fisher's exact test was used to examine associations between categorical variables.

Given our sample size of 27 participants per group, we were adequately powered to detect moderate to large effect sizes using two-sample *t*-tests and non-parametric equivalents (7). While this sample size may not be sufficient to detect small effect sizes, it is appropriate for identifying clinical meaningful differences and minimizing the likelihood that observed effects are due to random variation. Finally, given its standard clinical practice of not collecting post-group bloodwork when baseline values are within normal range for this clinic, ten imputations were applied to address missing data for cardiometabolic and BYI-2 variables.

## 3 Results

Of the 54 participants, both the virtual (*n* = 27) and in-person (*n* = 27) participants had a mean age of 12.26 years (Table 1), with each group composed of 78% boys and 22% girls. Over the 10-week intervention period, both groups experienced small decreases in BMI z-scores (Table 1), with no statistically significant differences between groups at baseline (*p* = 0.73) or post-intervention (*p* = 0.88).

**Table 1:** Population characteristics between virtual and in-person participants.

Variable	Virtual (*n*=27)	In- person (*n*=27)	*p*-value^1^	*p*-value^2^	Effect size	
Characteristics						
Age (years), mean (SD)^#^	12.26 (1.9)	12.26 (1.9)	1.00	–	0	–
Sex (%)*						
Male	21 (77.8)	21 (77.8)	1.00	–	–	–
Female	6 (22.2)	6 (22.2)			–	–
Anthropometrics						
Body mass index Z-score, mean (SD)						
Pre^$^	2.79 (0.8)	2.82 (0.5)	0.73	(0.87, 0.44)	0.05^$$^	0.03^##^, 0.14^##^
Post^$^	2.78 (0.8)	2.79 (0.5)	0.88		0.05^$$^	
Body mass index, mean (SD)						
Pre^#^	29.44 (3.9)	29.38 (2.6)	0.95	(**0.005**, 0.29)	0.02^##^	0.55^##^, 0.21^$$^
Post^$^	30.15 (4.0)	29.43 (2.7)	0.63		0.05^$$^	
Cardiometabolic measures^a^						
Triglycerides (mmol/L)						
Pre^$^	1.31 (0.8)	1.45 (1.0)	0.57	(0.53, 0.40)	0.16^$$^	0.16^##^, 0.17^##^
Post^$^	1.33 (0.6)	1.50 (0.9)	0.53		0.08^$$^	
Fasting glucose (mmol/L)						
Pre^$^	5.67 (1.7)	5.03 (1.0)	0.39	(**0.04**, 0.42)	0.18^$$^	0.48^$$^, 0.16^##^
Post^$^	5.03 (0.6)	5.02 (0.5)	0.33		0.25^$$^	
High-density lipoprotein (mmol/L)						
Pre^$^	1.13 (0.5)	1.27 (0.6)	0.53	(0.52, 0.46)	0.10^$$^	0.15^$$^, 0.17^##^
Post^$^	1.14 (0.8)	1.21 (0.6)	0.54		0.12^$$^	
Low-density lipoprotein cholesterol (mmol/L)						
Pre^#^	2.58 (0.9)	2.34 (0.8)	0.36	(0.54, **0.044**)	0.26^##^	0.13^##^, 0.52^##^
Post^#^	2.48 (1.1)	1.92 (1.0)	0.14		0.51^##^	
Total cholesterol (mmol/L)						
Pre^#^	4.15 (1.2)	4.14 (0.9)	0.65	(**0.03**, 0.23)	0.13^##^	0.46^##^, 0.35^##^
Post^#^	3.27 (1.5)	3.65 (1.6)	0.45		0.30^##^	
HDL-total cholesterol ratio						
Pre^#^	4.06 (1.9)	3.68 (1.5)	0.40	(0.28, 0.25)	0.24^##^	0.22^##^, 0.32^##^
Post^#^	3.85 (0.7)	3.41 (0.8)	0.11		0.55^##^	
Alanine aminotransferase enzymes (U/L)						
Pre^$^	25.76 (23.4)	42.88 (27.7)	0.06	(0.43, 0.055)	0.18^$$^	0.17^##^, 0.43^##^
Post^#^	27.55 (24.2)	31.06 (22.8)	0.43		0.23^##^	
Aspartate aminotransferase enzymes (U/L)						
Pre^$^	24.73 (14.3)	31.97 (15.5)	0.27	(0.46, 0.20)	0.23^$$^	0.16^##^, 0.27^$$^
Post^#^	22.72 (10.9)	24.57 (10.5)	0.50		0.19^##^	
Beck youth inventory						
Anxiety T-Score						
Pre^$^	48.36 (11.4)	49.47 (8.2)	0.41	(0.08, **0.01**)	0.16^$$^	0.38^$$^, 0.60^##^
Post^$^	45.35 (12.9)	44.78 (7.7)	0.77		0.11^$$^	
Depression T-Score						
Pre^$^	45.64 (9.9)	47.53 (7.2)	0.26	(0.37, **0.02**)	0.10^$$^	0.21^##^, 0.35^$$^
Post^$^	44.39 (12.3)	43.89 (7.9)	0.74		0.16^$$^	
Self-concept T-Score						
Pre^#^	47.84 (9.5)	47.98 (5.9)	0.91	(0.18, 0.44)	0.03^##^	0.29^##^, 0.14^$$^
Post^#^	50.97 (13.1)	49.70 (8.5)	0.66		0.12^##^	

a Reference norms: (1) Triglycerides = 0.40–1.29; (2) Fasting glucose = 3.3–6.0; (3) High-density lipoprotei*n* = >1.0; (4) Low-density lipoprotein = 1.50–2.79; (4) Total cholesterol = 2.00–4.39; (5) HDL-Total cholesterol ratio: <4.90; (6) Alanine aminotransferase enzymes = 10–35; (7) Aspartate aminotransferase enzymes = 10–60.

1 *p*-value comparing virtual and in-person groups.

# Parametric two-sample *t*-tests were used for numerical normally distributed characteristics. Significance was established at *p* < 0.05 and printed in boldface.

* Measure of association/independence determined any relation to the two categorical variables using the Fisher's exact test of independence.

$ The nonparametric test (Mann–Whitney *U*-test) were used to measure for a significant difference between the means of the virtual and in-person groups due to violating assumptions required for a parametric two-sample *t*-test of the variables.

2 *p*-value comparing pre- and post- measures (virtual, in-person). Pre- and post- measures for non-parametric data were compared by the Wilcoxon Signed-Rank Test.

## Effect size was measured using Cohen D for normally distributed data. Small effect, |r| is near 0.2; medium effect, |r| is near 0.5; large effect, |r| is near 0.8.

$$ Effect size was measured using the Mann–Whitney *U*-test (between groups) and Wilcoxon signed-rank test (within groups) for non-parametric data. Small effect, |r| is near 0.1; medium effect, |r| is near 0.3; large effect, |r| is near or exceeds 0.5.

There were no significant differences in pre- or post-intervention measures between the virtual and in-person groups for all cardiometabolic parameters; all were within normal clinical ranges (8, 9). At 10 weeks, in-person participants had mean triglyceride levels at the lower threshold of abnormal (≥1.5 mmol/LR) (10), while virtual participants had borderline levels (1.0 to <1.5 mmol/L). Fasting glucose levels improved from abnormal to normal (7) in the virtual group, decreasing from 5.67 mmol/L to 5.03 mmol/L, while remaining stable and within normal limits in the in-person group (5.03 mmol/L to 5.02 mmol/L; *p* = 0.42). Similarly, there were no significant differences in AST levels, despite a decrease in levels from baseline observed in both groups.

Effect size analyses revealed that post-intervention LDL and HDL ratio had the largest between-group effects, with Cohen's *d* values of 0.51 and 0.55, respectively (Table 1). These represent moderate effect sizes, although the differences were not statistically significant. All other between-group comparisons yielded small or negligible effect sizes.

Within-group analyses showed several significant changes. In the virtual group, BMI increased significantly (*p* = 0.005), with a moderate effect size of 0.55. Total cholesterol decreased significantly (*p* = 0.03), with a moderate effect size of 0.46, and fasting glucose decreased significantly (*p* = 0.04), with a large effect size of 0.48. In the in-person group, LDL levels decreased significantly (*p* = 0.044), with a moderate effect size of 0.52. ALT levels showed a near-significant increase (*p* = 0.055), with a moderate effect size of 0.43.

Finally, mental health outcomes of anxiety (*p* = 0.01) and depression (*p* = 0.02) scores decreased significantly, with moderate effect sizes of 0.60 and 0.35, respectively within the in-person group. There were no statistically significant differences in BYI-2T-scores between the virtual and in-person groups (all *p* > 0.05).

## 4 Discussion

This study examined the effectiveness of a virtual pediatric obesity management program implemented during the COVID-19 pandemic, and compared patient outcomes relative to the in-person program offered before the pandemic. We found no significant differences between the virtual and in-person groups in anthropometric measurements, cardiometabolic measures, or BYI-2 T-scores, suggesting that the virtual program was as effective as the in-person program in supporting patients.

Our results showed that both groups experienced decreases in BMI z-scores over the intervention period. However, these decreases were small and not statistically significant, likely due to the short duration of the program and its overall focus on health behavior change rather than weight loss. While the virtual group did experience an increase in BMI during the 10-week period, it is noteworthy that BMI is a poor indicator of adiposity in children—particularly when compared to age-adjusted norms such as BMI z-scores or percentiles. As BMI z-scores experienced a small decrease, it is likely that this observed increase in BMI is not reflective of an actual increase in adiposity during the intervention.

Our results for cardiometabolic measures are similar Chai et al.'s work (*n* = 36 families by 12-weeks), who found no significant difference in post-virtual interventions (11). While the majority of metabolic measures for participants in this study were within normal levels at baseline, the post-intervention values trended towards a more favorable profile, with the exception of triglycerides; a larger sample size is needed as we suspect this is a sample size artefact.

In this study, we report on changes in the BYI-2 questionnaire to assess mental health. An earlier study by Jacobson et al. (12) found no significant changes in 9–12 year-olds living with overweight and/or obesity in self-concept, anxiety, and depression in a 1-group, 1-week pre-/post-test study design. These null findings are likely due to the short trial length. When the in-person Shapedown BC program was assessed in 2011, Panagiotopoulos et al. (2) found significant improvements in participant self-concept and anxiety scores, but no improvements were seen in depression scores. Irrespective of findings, interventions that work with children and youth with overweight and obesity should include assessments of mental health (13), and screening and treatment for anxiety and depression, as they are imperative to support the quality of life in this population (14).

A key strength of this study was the inclusion of validated clinical mental health measures (BYI-2) alongside commonly studied outcomes (e.g., BMI, cardiometabolic indicators). This broader approach helps to better understand how virtual pediatric obesity management programs can impact both metabolic and mental health, beyond anthropometric changes. However, the study has several limitations. The 10-week program duration and absence of follow-up restrict the ability to assess long-term outcomes. The small sample size also limits generalizability, as it may not reflect the diversity of the pediatric population in British Columbia. A larger, more representative sample is needed to better capture the needs of this group. Additionally, the limited sample size prevented adjustment for baseline contextual differences, such as socioeconomic differences, between the virtual and in-person groups. Finally, COVID-19 social distancing measures in place during the study may have influenced participants’ health behaviours and affected the outcomes.

### 4.1 Conclusion

Our results suggest that the mode of program delivery (virtual or in-person) of a multidisciplinary pediatric obesity management team yielded similar outcomes by 10 weeks. Further research that investigates differences in participant sex and age group, with rigorous measures that ensure the accurate and complete collection of data is warranted.

## Data Availability

The raw data supporting the conclusions of this article will be made available by the authors, without undue reservation.
